# A New Model for Basic Microsurgical Nerve Repair Simulation: Making the Most Out of Less

**DOI:** 10.1055/s-0042-1758544

**Published:** 2023-02-10

**Authors:** Bogdan Ioncioaia

**Affiliations:** 1 Clinical Rehabilitation Hospital, Cluj-Napoca, Romania

**Keywords:** nerve repair simulation, epineurial repair, microsurgical training, microsurgery

## Abstract

Microsurgical peripheral nerve repair is a technical and challenging procedure that requires thorough training prior to a real-life operating theater scenario. While the gold standard in training remains training on biological living peripheral nerve specimen, various inanimate models of nerve repair simulation have been described in the past years.

The textile elastic band (TEB) obtained from a surgical mask was either covered with a fine silicone sheath or was left bare and was used afterward for end-to-end coaptation.

The average diameter of the TEB was 2 mm, similar with the nerves in the distal hand and can be easily crafted out of accessible materials such as a surgical mask and silicone sealant. The silicone that covers the TEB offers more fidelity to the simulation for microsurgical nerve coaptation.

The TEB model offers an affordable, available, and easy-to-craft alternative to the existing models for peripheral nerve repair simulation and serves as a good initiation tool before moving on to biological specimens.

The occurrence of peripheral nerve injuries is relatively frequent among the young adult population 1 carrying with itself the burden of increased financial expenses. 2

Due to their high incidence, the involvement of critical nervous structures, and the increased costs of treating peripheral nerve injuries, learning the proper technique of nerve repair needs to be pursued with great interest by the trainee to achieve the best results possible.

Nerve repair is usually taught in a microsurgical course on the rat model in the form of an epineurial repair of the sciatic nerve. 3 After acquiring the set of skills pertaining to microsurgical nerve coaptation, maintaining them is also important, as any other microsurgical skill set. 4

For the purpose of initiation in end-to-end coaptation, we propose the use of textile elastic band (TEB) before advancing toward more realistic scenarios such as on biological specimen.

A TEB was obtained from a surgical mask and was either covered with a fine silicone sheath ( Fig. 1A ) or left bare ( Fig. 1B ); the TEB was used to simulate end-to-end microsurgical coaptation ( Fig. 2 ). The microsurgical coaptation was performed after the TEB was sectioned with scissors, using a microscope or 2.5 to 3.5x magnification loupes with 8–0 nylon sutures.

**Fig. 1:**
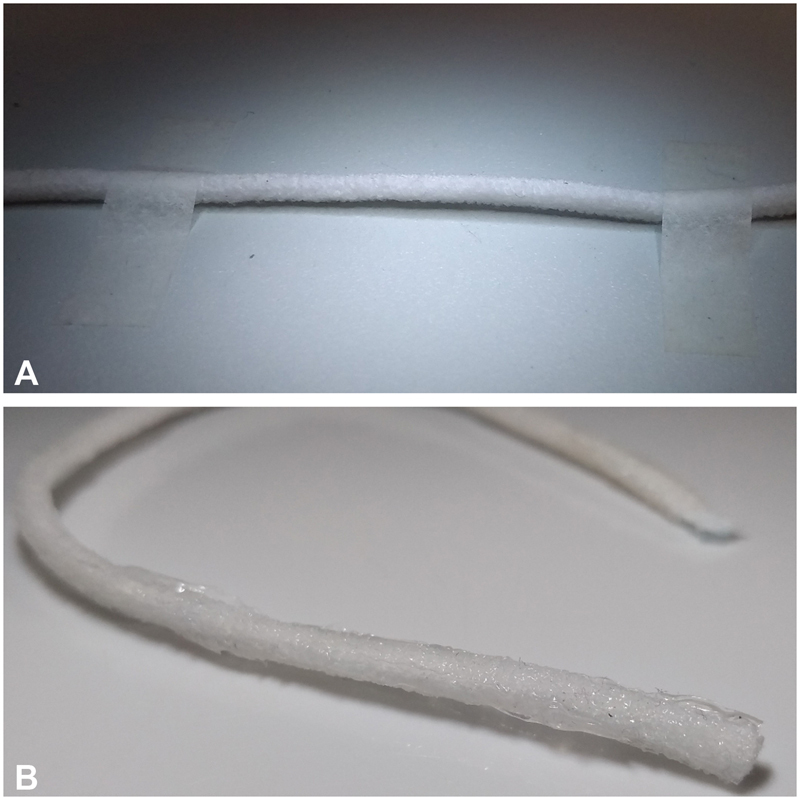
( **A** ) The bare textile elastic band (TEB) stretched on the worktable and fixed with medical tape. ( **B** ) The silicone-covered TEB.

**Fig. 2:**
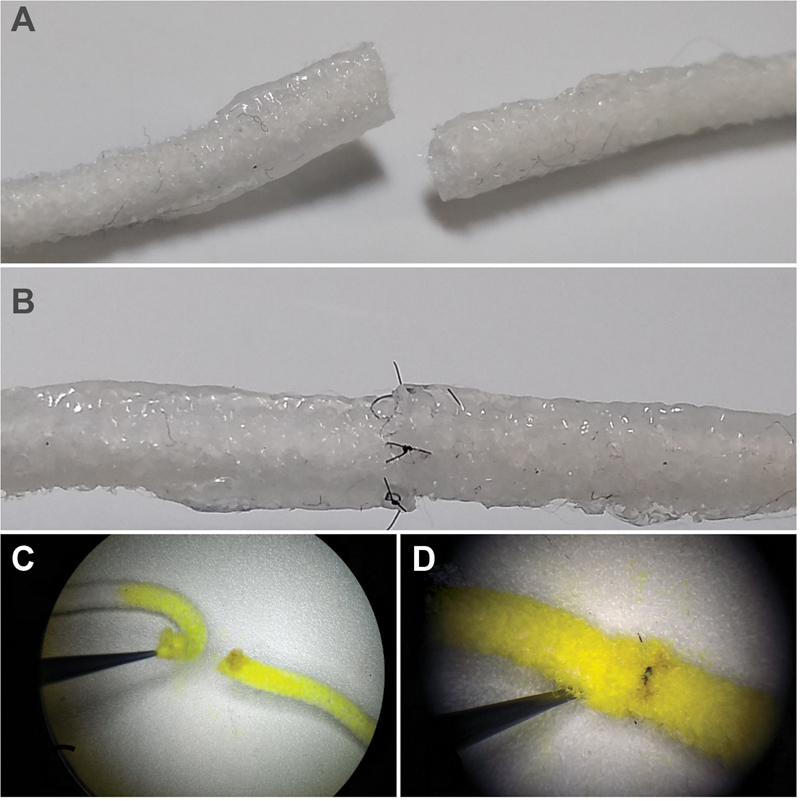
( **A** ) The aspect of the sectioned silicone-covered textile elastic band (TEB). ( **B** ) The repaired silicone-covered TEB. ( **C** and **D** ) The section and repair of a bare TEB as seen through the microscope.

The acquisition of microsurgical skills before going to the operating room was advocated since the pioneering days of microsurgery. 5 Various inanimate nerve repair models 6 7 8 9 have been described that could be used as simulators and with the expectancy to improve technical performance, similar to microvascular repair simulators. 4 Senturk et al described a training model manufactured out of multiple rubber threads covered by Steristrip, 6 rendering it suitable for fascicular suture, nerve graft, and nerve transfer training. The disadvantage of this model is its difficulty in constructing it and the low fidelity for performing epineurial repair. Shah et al described a training model build up from a piece of twine rolled in a cling film used for epineurial repair, 7 but due to its torsion, it loses its fidelity when considering the structure it must mimic. Kamath et al described a model consisting of sterile surgical disposable hand towels filled with different hollow plastic tubes imitating a fascicular arrangement contained within an epineurial sheath with the possibility to simulate fascicular repair. 8 However, the surgical towel is less suitable for epineurial repair due to its firmness. Gul et al described a silicone-based simulation model manufactured after different formulations with increased fidelity, 9 but this model was built using a technological advanced process, rendering it impossible for self-manufacture, compared with the previously mentioned models.

The TEB model can be easily crafted out of accessible materials such as a surgical mask and silicone sealant. The silicone that covers the TEB offers more fidelity to the simulation, due to its function as an epineurial sheath imitation, aiding the placing of epineurial and even epineurial sutures thanks to its decreased opacity. Additional procedures such as a nerve graft or nerve transfer simulation may also be attempted. The average diameter of the TEB was 2 mm, similar with the nerves in the distal hand. Preparing the TEB for simulation can be done in less than 5 minutes if used without silicone covering and in less than 1 hour if using silicone. The silicone covering and the use of microsurgical instrumentation and magnification offer precision and thus an increased fidelity for epineurial nerve repair. Magnification loupes (2.5x), microsurgical forceps, needle holder, and an 8–0 suture will suffice for basic simulation in the absence of a microscope. From an economical standpoint, the cost of production per model is less than 0.5 US dollar or euro. The TEB model offers the advantage of low cost, availability, easy manufacturing, and the possibility of its use in an “dry,” home-friendly environment and serves as a good initiation tool before moving on to biological specimens.
